# Medication-Induced Xerostomia: Cross-Sectional Analysis of Salivary Flow, Intraoral Aching, and Anxiety

**DOI:** 10.3390/jcm14186624

**Published:** 2025-09-19

**Authors:** Olga A. Korczeniewska, Eli Eliav, Szilvia Arany

**Affiliations:** 1 Center for Orofacial and Temporomandibular Disorders, Department of Diagnostic Sciences, Rutgers School of Dental Medicine, Rutgers, The State University of New Jersey, Newark, NJ 07103, USA; korczeol@sdm.rutgers.edu; 2 Eastman Institute for Oral Health, University of Rochester, Rochester, NY 14620, USA; 3 Specialty Care, Eastman Institute for Oral Health, University of Rochester, Rochester, NY 14620, USA

**Keywords:** xerostomia, medication, saliva flow, intraoral painful aching

## Abstract

**Background:** This cross-sectional study investigated the associations between medication-induced xerostomia (perceived oral dryness) and intraoral painful aching in 141 middle-aged adults (45–64 years) with self-reported xerostomia resulting from anticholinergic medications. **Methods:** Xerostomia severity, anxiety, and intraoral painful aching were evaluated using questionnaires, including the semiquantitative Xerostomia Inventory survey. Reduction in saliva secretion (hyposalivation) was objectively assessed by the measurement of unstimulated whole saliva (UWS) flow. **Results:** Multivariate stepwise linear regression was used to identify factors associated with XI scores, adjusting for potential confounders including age, sex, diabetes, smoking status, and race. The final model identified UWS flow (*p* = 0.0023), intraoral painful aching (*p* = 0.0030), and diabetes (*p* = 0.0097) as significant predictors of xerostomia severity. Anxiety demonstrated a marginal association (*p* = 0.0643) and accounted for a smaller proportion of model variance. Relative importance analysis revealed that UWS flow contributed 33.16% to the overall model fit, followed by intraoral pain (31.30%), diabetes (23.60%), and anxiety (11.93%). **Conclusions:** The findings indicate that reduced salivary flow, intraoral discomfort, and the presence of diabetes are significant contributors to xerostomia severity in individuals taking anticholinergic medications. These results highlight the importance of individualized evaluation in xerostomia care and inform targeted clinical strategies for managing xerostomia symptoms in patients with intraoral painful aching, anxiety, or comorbid diabetes.

## 1. Introduction

Xerostomia, the subjective sensation of oral dryness, affects approximately 25 million individuals in the United States [1,2]. Numerous medications, such as antiparkinson agents, antispasmodics, antidepressants, antipsychotics, and cold and allergy remedies, exhibit anticholinergic side effects that disrupt parasympathetic cholinergic signalling, impairing the normal function of the salivary glands [3,4]. Trends in medication use indicate a 70% increase in polypharmacy involving five or more medications, alongside a significant rise in anticholinergic exposure since 1995 [5,6]. The prevalence of medication-induced xerostomia is estimated to range between 12% and 39% in both the US and globally [7]. Xerostomia [8] may manifest with or without hyposalivation, a clinically measured decrease in saliva secretion. Hyposalivation is clinically assessed by measuring the unstimulated whole saliva (UWS) flow rate, which remains the standard test for evaluating changes in saliva production [9,10].

Widely recognized confounding factors, including aging and biological sex, influence the symptoms of hyposalivation and xerostomia. The prevalence of xerostomia in adults varies widely, ranging from 14% to 46%, with women being more frequently affected [1,11,12]. Additionally, women are associated with higher medication use, greater anticholinergic exposure, reduced saliva flow rates, and increased reports of xerostomia [13]. Recent studies involving cancer and diabetic populations revealed that females have lower salivary flow rates compared to males, resulting in an elevated risk of xerostomia [14,15,16].

Clinical reports consistently correlate xerostomia with intraoral discomfort and aching. Xerostomia and hyposalivation compromise the moisturization of the oral cavity, leading to significant dental, oral, and craniofacial issues, such as discomfort, mucositis, ulcers, and pain [17]. Individuals experiencing dry and sore mouths face considerable difficulty eating and maintaining oral health. Chronic hyposalivation caused by medications frequently leads to irritation, cracking, and inflammation of the oral mucosa, thereby intensifying intraoral discomfort [18]. Psychological factors, such as heightened anxiety, often occur with xerostomia, as stress-related inhibition of the parasympathetic nervous system may further reduce saliva secretion, which may exacerbate intraoral discomfort [2,19]. In a systematic review, we synthesized the existing scientific evidence from available publications on the relationship between xerostomia and intraoral pain in patients using medications, highlighting the scarcity of research examining this connection, underscoring the need for targeted studies [17]. The review also emphasized the limited evidence on the pharmacological classification and characteristics of medications responsible for xerostomia and orofacial pain, and recommended considering oral pain as a potential clinical predictor of medication-induced oral health damage While the relationship between xerostomia and chronic pain in the orofacial region, such as temporomandibular pain, headache, and masticatory abnormalities [20], is commonly discussed in the literature, the discomfort and aching in the mouth due to xerostomia remains less reported, despite its frequent clinical prevalence. In this cross-sectional study, we aimed to examine the associations between medication-induced xerostomia and intraoral painful aching, and to assess the relationship between drug-induced hyposalivation and the severity of xerostomia.

## 2. Materials and Methods

**Figure 1:**
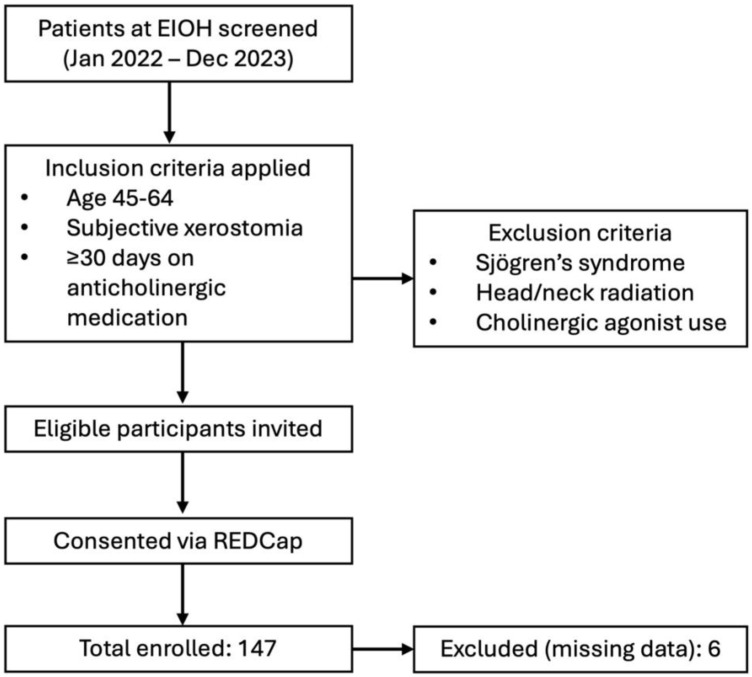
**Participant enrollment flowchart**. Study recruitment from screening to final participant inclusion, showing application of eligibility criteria and exclusions. (EIOH; Eastman Institute for Oral Health).

Recruitment: The study was conducted according to the guidelines of the Declaration of Helsinki and approved by the University of Rochester Medical Center (URMC) Institutional Review Board (RSRB STUDY00005666) on 15 June 2021, in compliance with Federal regulation 45 CFR 46 under the University’s Federal-wide Assurance (FWA00009386). A detailed flowchart depicting participant recruitment, eligibility screening, exclusion criteria, and final enrollment steps is presented in Figure 1. Participants for this study were recruited from adults visiting the Specialty Care Clinic in General Dentistry at the Eastman Institute for Oral Health (EIOH) between 01/2022 and 12/2023. The study investigators enrolled individuals who expressed interest and met the eligibility criteria after obtaining informed consent. Informed consent was obtained from all subjects involved in the study. Eligibility information was accessed through EIOH Axium records (dental charts) and URMC electronic medical records. During their study visit, participants submitted electronic consent forms and study questionnaires via the University of Rochester Research Electronic Data Capture (REDCap) platform. A sample size calculation based on reported mean UWS values in medication-evoked xerostomia showed that a sample size of 125 achieves 80% power to detect a significant difference at a significance level of 0.05. As the highest incidence of medication-induced xerostomia (28%) was recorded in the middle-aged adult population in a recent meta-analysis [19], we recruited individuals from this age group. We enrolled 147 middle-aged adult patients aged 45–64 in this study.

**Eligibility criteria** (inclusion): 1. Age between 45 and 64 years. 2. Complaint of oral dryness (xerostomia). 3. Continuous use of at least one anticholinergic medication for the past 30 days or longer, prescribed for long-term use (verifiable through electronic health records or a provider’s note). We utilized the modified (updated and dose-weighted) anticholinergic drug scoring, which includes 536 medications [21,22], to identify those with anticholinergic potential. Individuals were excluded if they were diagnosed with Sjögren’s syndrome or other diseases affecting the salivary glands, had a history or current treatment with head-and-neck radiation therapy or radioiodine, or were currently using cholinergic agonists. Self-administered questionnaires were used to collect demographic information (age, sex, race). Comorbidities such as smoking, hypertension, depression, and diabetes, which often correlate to hyposalivation, were recorded from electronic medical charts and collected through the University of Rochester REDCap platform, a secure, web-based software platform designed to support data capture for research studies.

**Saliva flow assessments:** Resting saliva secretion from all salivary glands was measured using the UWS flow rate. To mitigate circadian fluctuations, appointments were scheduled between 9:00 a.m. and 12:00 p.m. Participants were instructed to refrain from eating, drinking, smoking, brushing their teeth, using mouthwash, chewing gum, or rinsing their mouths with water for at least one hour before their visits. Saliva was collected by the “spitting” method [23] in pre-weighed plastic tubes over a 10 min period while participants sat upright. We selected the spitting technique based on established salivary collection protocols, particularly in populations with reduced salivary flow [9,24]. Subjects were instructed to spit into the collection tube, and the saliva volume [25] was determined gravimetrically using a calibrated analytical balance. Saliva volume was calculated using the assumption of 1.0 g/mL saliva density, and then flow rates were expressed in mL/min. Hyposalivation was confirmed when the UWS volume was ≤0.1 mL/min [8,26].

**Xerostomia assessment:** The subjective perception of oral dryness was assessed using the Xerostomia Inventory, a validated English-language questionnaire, widely used in US clinical and research settings [27]. This questionnaire evaluates xerostomia on a continuous scale via 11 questions, each scored on a 5-point Likert scale. Total scores range from 11 to 55, with higher scores indicating greater severity of xerostomia.

**Anxiety and intraoral pain assessment:** Anxiety levels were assessed using a single-item visual analog scale [28], where participants rated their perceived anxiety from 0 (no anxiety) to 10 (worst possible anxiety). This simple measure, previously applied in dental and orofacial pain settings [28,29] was selected for its efficiency and feasibility in a clinical research environment, although it does not capture the full scope of anxiety symptoms. The frequency of painful aching in the mouth was evaluated using a 5-point Likert scale with the following levels: “Never” (score 0), “Hardly Ever” (score 1), “Occasionally” (score 2), “Fairly Often” (score 3), and “Very Often” (score 4) [30].

### Statistical Analysis

Statistical analyses were performed using JMP^®^ Pro 18.0.2, Microsoft Windows 10 Enterprise (10.0.19045.0) software. The primary analytical framework was based on the following hypotheses. Alternative hypothesis (H_1_): medication-induced xerostomia is significantly associated with increased intraoral aching, anxiety, and reduced salivary flow, and these associations vary with other variables, such as comorbidities, smoking status, age, race, and biological sex. Null hypothesis (H_0_): no significant associations exist between medication-induced xerostomia, intraoral aching, anxiety, and salivary flow. These hypotheses were evaluated using a multivariable linear regression analysis to identify statistically significant relationships while adjusting for relevant covariates.

Forward stepwise linear regression analysis was performed using a minimum AIC stopping rule to reduce the number of variables included in the model and identify those significantly influencing XI (the dependent variable). For categorical variables with more than two levels, the whole effects rule was applied. This approach enters all levels of the variable into the model together as a single effect, rather than testing each level separately. Post hoc comparisons were performed using Student’s *t*-tests of least-squares means (LSMeans) for the significant variables. A significance level of alpha < 0.05 was considered statistically significant. To assess the relative contribution of each predictor to the regression model, we calculated predictor importance using normalized F-ratios. These F-ratios were obtained from the model’s effect tests and reflect the unique variance explained by each predictor when entered last into the model. Each F-ratio was divided by the total sum of all F-ratios to produce a proportion representing the relative influence of each variable. This method allows comparison of predictors measured on different scales. Multicollinearity in the regression analysis was identified using variance inflation factors (VIFs) that quantify the severity of multicollinearity for each variable in a model.

## 3. Results

**Figure 2:**
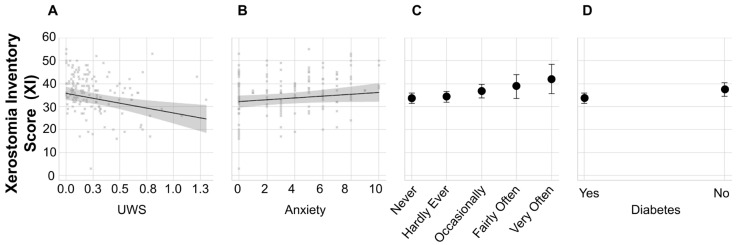
**Predictors of Xerostomia Inventory Score.** Xerostomia scores are shown across levels of significant and near-significant predictors from the multiple linear regression model. (**A**) Xerostomia scores decrease with increasing unstimulated whole saliva flow (UWS). (**B**) A modest positive trend is observed between anxiety and xerostomia. (**C**) Xerostomia scores increase progressively with the frequency of painful aching in the mouth. (**D**) Individuals with diabetes exhibit higher predicted xerostomia scores compared to those without diabetes. Prediction profiler graphs visualize how changing individual predictors affects the predicted response in a multiple regression model. The red lines correspond to profile traces for each predictor, showing the change in predicted response as one variable is varied while others are held constant at their current values. The slope of each line reflects the coefficient and significance of the predictor in the model. A steeper slope indicates a stronger effect on the predicted response. Prediction intervals are represented as curves (for continuous factors) and as error bars (for categorical factors). Figure 3. Relative contribution of each predictor to the Xerostomia Inventory score based on a multivariate linear regression model.

**Figure 3:**
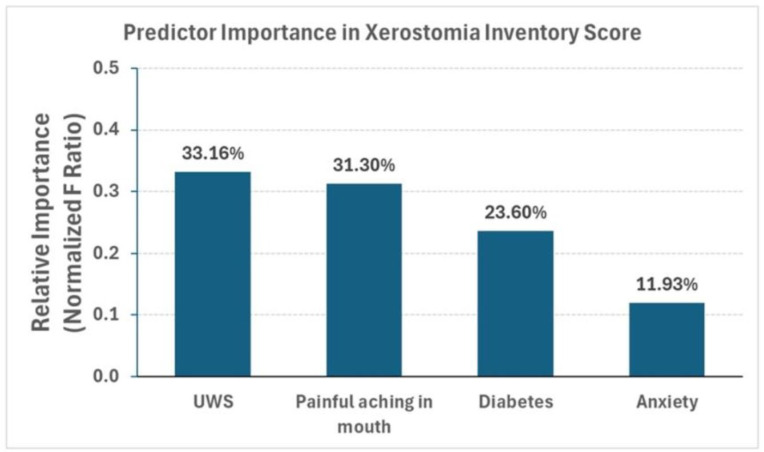
**Predictor Importance in Xerostomia Inventory Score Regression Model.** The bar graph illustrates the importance of predictors in the regression model of XI. The importance was calculated using normalized F-ratios. Unstimulated whole saliva flow (UWS) was the most significant predictor of XI (33.16%), followed closely by the presence of painful aching in the mouth (31.30%). Diabetes contributed moderately (23.60%), and anxiety had a relatively low influence on XI (11.93%). UWS = Unstimulated whole saliva flow.

Demographic data are summarized in Table 1. One hundred and forty-one participants were included in the analysis, with six participants excluded due to missing data. A multivariate forward stepwise linear regression analysis was conducted to identify factors associated with the Xerostomia Inventory score. The independent variables initially included in the model were unstimulated whole saliva (UWS) flow, anxiety, and intraoral painful aching in the mouth. To account for potential confounding, age, biological sex, diabetes status, smoking status (current, former, or never), and race were also included in the analysis. The final model identified three variables that were significantly associated with XI scores: UWS flow (*p* = 0.0023), painful aching in the mouth (*p* = 0.0030), and diabetes (*p* = 0.0097) (Table 2, Figure 2). Anxiety demonstrated a marginal association with XI scores (*p* = 0.0643). In contrast, age, sex, smoking status, and race were not significantly associated with XI and were excluded from the final model. The analysis of collinearity between the included predictor variables, using variance inflation factors (VIFs), revealed no correlation between any of the variables, as indicated by VIFs of approximately one for each variable (Table 2). Our results revealed a negative relationship between UWS flow and XI index. Additionally, higher XI scores were significantly associated with an increased frequency of self-reported painful aching in the mouth and the presence of diabetes. Individuals who reported experiencing intraoral pain “very often” presented with a higher XI score compared to those who reported “never” experiencing painful aching in the mouth. Finally, a modest positive trend was observed between anxiety and XI severity. We computed the relative importance of each predictor of the XI score using normalized F-ratios (Figure 3). Based on our analyses, UWS was the most significant predictor of XI, accounting for 33.16% of the overall model fit, followed closely by the presence of painful aching in the mouth, which contributed 31.30%. Diabetes had a moderate contribution of 23.60%, and anxiety had a relatively low influence on XI, contributing 11.93% to the overall model, after accounting for the effects of other predictors.

## 4. Discussion

Our cross-sectional investigation identified significant associations between medication-induced xerostomia and multiple variables, including unstimulated whole saliva flow (UWS), intraoral painful aching, anxiety, and diabetes. These findings suggest that both physiological (e.g., reduced UWS flow, diabetes) and subjective (e.g., perceived oral discomfort, anxiety) factors contribute to the severity of xerostomia. However, unexplained variance in our model suggests that additional variables, such as genetic or neurobiological mechanisms, may influence xerostomia presentation.

We found a significant association between xerostomia and painful aching in the mouth, as individuals reporting frequent oral pain indicated higher xerostomia scores compared to those with infrequent or no intraoral pain. This finding confirms the observations reported by Ship [31] about correlations between dry mucosal surfaces and heightened oral sensitivity or discomfort. Medication-induced xerostomia and/or hyposalivation often acts as a trigger for secondary orofacial pain, necessitating comprehensive management strategies that address both dryness and associated discomfort. This relationship highlights the impact of medications, particularly those with anticholinergic effects, on oral health and quality of life, as hyposalivation often exacerbates mucosal irritation, leading to discomfort and secondary pain, as previously described by Sreebny and Schwartz [3]. The bidirectional link between xerostomia and oral discomfort suggests that addressing xerostomia may alleviate associated oral pain and vice versa. Miranda-Rius et al. [18] supported this idea, emphasizing that therapeutic approaches targeting xerostomia often reduce mucosal irritation and related intraoral pain, necessitating the development of tailored interventions. The strength of this association from our investigation indicates a need for clinical attention to self-reported intraoral pain when evaluating xerostomia severity. Therefore, future studies should explore integrated therapies that address both xerostomia and intraoral pain, aiming to potentiate synergistic benefits.

Anxiety showed a marginal association with xerostomia scores in our analysis, contributing a relatively small portion of the explained variance. This potential association between xerostomia and anxiety highlights the psychological dimension of xerostomia and the bidirectional relationship between psychological well-being and salivary gland function—chronic stress and anxiety may alter autonomic nervous system regulation [32], contributing to reduced salivary flow and heightened xerostomia perception [33]. Moreover, individuals reporting frequent painful aching in the mouth also exhibited elevated anxiety levels, suggesting that oral pain may exacerbate psychological distress, further compounding anxiety, particularly in individuals experiencing such pain frequently. These results are reinforced by the earlier findings [34] that intraoral pain aggravates emotional distress, suggesting that xerostomia, oral discomfort, and anxiety form a mutually reinforcing triad [35]. While not statistically significant, the observed trend supports the need to further examine the psychological aspects of xerostomia in future studies.

Additionally, xerostomia management strategies that integrate psychological support with physical symptom relief could benefit affected individuals.

Study participants with diabetes presented significantly higher xerostomia scores compared to non-diabetic individuals, suggesting that diabetes is an independent contributor to the perceived severity of medication-induced xerostomia. Hyperglycaemia-induced alterations in salivary gland microvascular function and neuropathy may contribute to reduced salivary flow and heightened perception of xerostomia in this population [36]. Similar conclusions were drawn by Zieba et al. [37] that smokers often continue to experience symptoms of xerostomia due to alterations in saliva composition and quality.

This study was limited by its cross-sectional design, which precludes causal inferences and a relatively small cohort size. Temporal relationships between variables could not be determined, and associations should be interpreted cautiously. The utilization of self-reported questionnaires assessing xerostomia, anxiety and intraoral aching introduces potential bias due to subjective variations in symptom perception. To mitigate the potential information bias, we used validated instruments for subjective scales (e.g., anxiety, oral discomfort). However, the anxiety scale was limited to a single-item visual analog measure, which does not capture the full spectrum of anxiety symptoms; future research should employ multidimensional tools. Although we mitigated potential bias due to confounding by using multivariable regression models to adjust for known and suspected confounders and included interaction terms when biologically possible, other potential confounders, such as medication dosage and utilization history, glycemic control, eating disorders, or aphagia, and mental health history, may influence the observed associations. Another source of bias may be due to outfitting, which occurs when including too many predictors in regression models relative to the sample size, leading to unstable estimates. To mitigate potential bias due to outfitting, we employed model selection strategies, including stepwise regression with AIC criteria. Additionally, the study sample was derived from a single clinical site, restricted to middle-aged adults, which may reduce the generalizability of the findings to broader populations. Finally, our study does not provide insights into qualitative aspects of saliva and saliva composition, which should be prioritized in future investigations. We must emphasize that the collection of saliva from minor mucosal glands should be cautiously interpreted due to methodological inconsistencies and varying locations in the oral cavity.

Our findings may have clinical relevance for the management of xerostomia. Management approaches should consider the interplay between xerostomia, intraoral aching, and discomfort to improve patient outcomes and quality of life. Our findings underscore the importance of individualized evaluation in xerostomia care, including targeted screening and management strategies for anxiety and oral discomfort that are essential. Particularly, screening for diabetes and anxiety in patients with medication-induced xerostomia may help identify underlying systemic or psychological factors. Future research should investigate the molecular and biological mechanisms underlying salivary function, oral pain, and diabetes in relation to the severity of xerostomia. Interventional studies addressing anxiety and intraoral discomfort in affected patients may yield insights into improving care. Our findings underscore the multifactorial nature of medication-induced xerostomia and its relationship with systemic health and psychological factors.

## 5. Conclusions

This study highlights the complex associations between medication-induced xerostomia as measured by the xerostomia inventory score and systemic as well as psychological variables. Lower UWS flow rates, more frequent painful aching in the mouth, and the presence of diabetes were significantly associated with an increased xerostomia inventory score. Higher levels of anxiety marginally contributed to increased xerostomia inventory score. These findings are based on a population of middle-aged adults using anticholinergic medications and should be cautiously extrapolated to other groups. Understanding these associations may support targeted screening of patients with diabetes and intraoral discomfort and guide hypothesis generation for future research aimed at improving xerostomia management.

## Figures and Tables

**Table 1:** Demographic data summary table.

	Total	Female	Male
	Mean ± S.D. (Min, Max), N	Mean ± S.D. (Min, Max), N	Mean ± S.D. (Min, Max), N
**Age**	55.86 ± 5.73 (45, 64), 147	55.81 ± 5.72 (45, 64), 104	56.00 ± 5.80 (45, 64), 43
**Race (N)**	Asian (1)	Asian (1)	Asian (0)
	Black (25)	Black (17)	Black (8)
	Native American (10)	Native American (4)	Native American (6)
	Other (6)	Other (4)	Other (2)
	Prefer not to answer (2)	Prefer not to answer (2)	Prefer not to answer (0)
	White (103)	White (76)	White (27)
	Mean ± S.D.	Mean ± S.D.	Mean ± S.D.
**Xerostomia Score**	36.29 ± 8.17	37.36 ± 7.70	33.70 ± 8.78
**Painful Aching in the Mouth (N)**	Never (55)	Never (39)	Never (16)
	Hardly Ever (47)	Hardly Ever (28)	Hardly Ever (19)
	Occasionally (29)	Occasionally (22)	Occasionally (7)
	Fairly Often (10)	Fairly Often (10)	Fairly Often (0)
	Very Often (6)	Very Often (5)	Very Often (1)
**Smoking (N)**	Yes, current (28)	Yes, current (18)	Yes, current (10)
	Former (52)	Former (36)	Former (16)
	Never (67)	Never (50)	Never (17)
**Diabetes (N)**	Yes (38)	Yes (26)	Yes (12)
	No (109)	No (78)	No (31)
	Median (IQR)	Median (IQR)	Median (IQR)
**Minor Saliva Flow (MSF) (ml/min)**	6 (3.5, 8)	5 (3.5, 8.0)	7 (5, 10)
**Unstimulated Whole Saliva Flow (UWS) (ml/min)**	0.19 (0.07, 0.32)	0.16 (0.06, 0.28)	0.25 (0.14, 0.60)
**Anxiety**	3 (0.75, 6)	4 (2, 6)	3 (0, 4)

**Table 2:** Significant predictors of xerostomia index score in stepwise linear regression final model.

Predictor Variable	Estimate	Std Error	F Ratio	t Ratio	Prob > |t|	Lower 95%	Upper 95%	VIF
**UWS**	−8.50	2.73	9.66	−3.11	0.0023	−13.90	−3.09	1.04
**Anxiety**	0.42	0.22	3.48	1.86	0.0643	−0.02	0.86	1.11
**Painful Aching in the Mouth**	1.83	0.61	9.12	3.02	0.0030	0.63	3.03	1.11
**Diabetes [No]**	−1.93	0.74	6.88	−2.62	0.0097	−3.38	−0.47	1.04

VIF = Variance inflation factors. VIF quantifies the severity of multicollinearity for each variable in the model. VIF of 1 indicates no multicollinearity.

## Data Availability

The data presented in this study are available on request from the corresponding author due to the NIH data sharing policy.
